# Prevalence of Staphylococcus aureus, Escherichia coli and Salmonella spp. isolated from meat and cooked meat at Khon Kaen Municipality Schools

**DOI:** 10.1186/2047-2994-4-S1-P114

**Published:** 2015-06-16

**Authors:** A Polpakdee, S Angkititrakul

**Affiliations:** 1 Veterinary Public Health, Khon Kaen University, Khon Kaen, Thailand

## Introduction

*Staphylococcus aureus*, *Escherichia coli* and *Salmonella* spp. are important causes of enteric illness. Foods of animal origin have been consistently implicated as the main sources of enteritis in school children.

## Objectives

The purpose of this study was to determine the prevalence of *Staphylococcus aureus*, *E. coli* and *Salmonella* spp. in meat and cooked meat.

## Methods

The raw and cooked pork and chicken in the canteens from 11 Khon Kaen Municipality Schools (average age 10-16 year) were collected between February and March 2013. Fifty-three were from raw meat and 91 were from cooked meat. *Salmonella* spp., *Staphylococcus aureus* and *Escherichia coli* were detected by ISO 6579:2002, AOAC Official Method 998.08 (3M Petrifilm) and AOAC Official Method 2003.11 (3M Petrifilm), respectively.

## Results

The results showed that the raw meats were contaminated with *S. aureus*, *E.coli* and *Salmonella* spp. 43.40%, 62.26%, 56.60% and 25.27%, 43.96%, 7.69% in cooked meats, respectively.

## Conclusion

The source for meat consumption should be from standard farms and slaughterhouses. In addition, hygienic kitchens, cooking skills, and healthy cooks are major factors for hygienic municipality school canteens, so that they are safe for school children consumption.

## Disclosure of interest

None declared.
